# Psychological stress and semen quality impairment: A systematic review

**DOI:** 10.5935/1518-0557.20260023

**Published:** 2026

**Authors:** Ane Francyne Costa, Lucas Valentim Silveira, Fabiana Botelho de Miranda Onofre, Alexandre Sherlley Casimiro Onofre

**Affiliations:** 1 Department of Clinical Analysis, Federal University of Santa Catarina, Florianópolis, SC, Brazil

**Keywords:** semen quality, psychological stress, sperm analysis, male infertility

## Abstract

**Objective:**

The aim of this review was to evaluate the extent to which psychological stress may adversely affect seminal quality parameters in men.

**Methods:**

A systematic review was conducted across databases including Embase, PubMed, Scopus, and Web of Science. Studies were subjected to data extraction, risk of bias, and narrative synthesis.

**Results:**

A total of 34 studies were included in this review. Three (8.82%) studies were judged to be at a high risk of bias. Included studies were published between 1988 and 2023 and concerned a total of 11,141 participants, ranging from 20 to 1,362 participants per study. Psychological stress negatively impacted semen volume in 12.9% of studies, sperm concentration in 52.9%, total sperm count in 56.3%, sperm motility in 63.3%, and sperm morphology in 21.4%. Sources of psychological stress such as academic stress, daily life stress, infertility-related stress, stressful life events, and work-related stress were correlated to impairment of semen parameters in 70.6% of included studies. The ability to draw robust conclusions is severely restricted by significant heterogeneity across the reviewed literature.

**Conclusions:**

This systematic review found that psychological stress is frequently associated with impaired semen parameters, particularly sperm concentration and sperm motility. Nevertheless, these findings should be interpreted with caution. Most of the reported associations are observational and do not establish a definitive causal relationship between psychological stress and semen quality impairment.

## INTRODUCTION

Infertility is defined as the absence of pregnancy after one year of regular unprotected sexual intercourse (Zegers-Hochschild *et al*., 2017). This clinical condition impacts approximately 45.8 million couples globally and has garnered attention due to declining fertility rates in numerous developed nations and the rising prevalence of in vitro fertilization (IVF) treatments (Vander Borght & Wyns, 2018; Schmidt *et al*., 2012). Infertility may arise from male factors, female factors, or a combination of both. In general, approximately 30% of infertility instances can be attributed solely to male factors (Agarwal *et al*., 2015).

Psychological stress has been correlated with a decline in semen quality parameters throughout the years; however, the findings vary significantly and present contradictory outcomes (Bhadoria *et al*., 2020; Nordkap *et al*., 2020). One of the primary biological rationales for the correlation between psychological stress and diminished semen quality pertains to fluctuations in the secretion of hormones from the hypothalamic-pituitary-gonadal axis in reaction to stress, which subsequently modifies the functionality of Sertoli cells and their associated blood-testis barrier (Bhongade *et al*., 2015; Nargund, 2015).

Conversely, it is challenging to delineate psychological stress as a standalone and principal contributor to male infertility, given that confounding variables like obesity, alcohol intake, and tobacco usage frequently correlate with individuals experiencing depression and anxiety (Regier *et al.,* 1990; AL-Asadi *et al.,* 2015). Male reproductive health is influenced by a wide range of factors including paternal age, nutrition, physical activity, obesity, caffeine intake, scrotal temperature, clothing choices, heat exposure, and mobile phone use, all of which can affect semen parameters and DNA vulnerability to reactive oxygen species (ROS) (Ilacqua *et al.*, 2018). Although psychological factors are linked to infertility, causal relationships remain difficult to establish. While various forms of stress (physical, emotional, or biological) may reduce male fertility potential, there is still no consensus on how to measure stress objectively. Consequently, it is imperative that confounding variables are considered in order to accurately ascertain the correlation between psychological stress and seminal parameters.

There are a multitude of questionnaires that can be utilized to assess psychological stress among males; nevertheless, it remains unclear which instrument has the highest sensitivity for identifying psychological stress, which is significant enough to potentially induce adverse effects on testicular function. Besides that, employment of a myriad of study designs can make direct comparison between studies a challenge. Consequently, the aim of this review was to evaluate the extent to which psychological stress may adversely affect seminal quality parameters in men.

## METHODS

This systematic review was conducted in accordance with guidelines established by the Preferred Reporting Items for Systematic reviews and Meta-Analysis (PRISMA) statement (Page *et al*., 2021). The review protocol was registered with the International Prospective Register of Systematic Reviews (PROSPERO), registration number CRD42021240164 (Costa *et al*., 2024).

### Eligibility criteria

This review was performed to address the following question: Does psychological stress impair semen quality parameters in men?

Full-report cross-sectional and longitudinal studies were considered. Studies were eligible if they included men who answered validated questionnaires to assess psychological stress and simultaneously provided a semen sample for analysis of their semen quality parameters. Using only validated questionnaires ensured that studies were measuring the same phenomena with similar precision, improving comparability between studies and reducing the risk of measurement bias.

The exclusion criteria were designed to exclude studies that did not meet the study main questions, including: studies that failed to evaluate both psychological factors and semen quality parameters; use of non-validated questionnaires to assess psychological stress; studies in which semen parameters were not assessed; studies without abstracts; abstract-only reports; non-human studies; case reports; letters; and comments.

Grey literature and unpublished studies were not included to avoid bias due to limited or absent peer review and to ensure methodological rigor, transparency, and feasibility.

### Information sources and search strategy

Comprehensive searches of published article data related to the study question were conducted in Embase, PubMed, Scopus, and Web of Science databases. Relevant additional literature was also identified through quotation of selected articles. A preliminary search was conducted to identify relevant terms to ensure a thorough and precise retrieval of eligible studies. The following keywords were used in the searches: (“Psychological stress” OR “Stressful life events” OR “Perceived stress”) AND (“Semen Analysis” OR “Semen quality” OR “Semen parameters” OR “Sperm count” OR “Sperm motility” OR “Sperm morphology” OR “Sperm vitality” OR “Male infertility”).

No language constraint was used during the selection process. Studies in which semen quality parameters were not assessed in accordance with any of the six editions of the World Health Organization laboratory manual for the examination and processing of human semen (Wang *et al*., 2022) were not considered, limiting inclusion of studies published only after 1980 when the first edition of the manual was released.

### Selection and data collection process

All quotations retrieved in the database searches were reviewed independently by two authors (AFC and LVS) for relevance based on titles and abstracts. Full text of potentially relevant studies was then screened against the inclusion criteria and accepted or rejected, as appropriate. Any disagreements in individual judgments of the reviewers were solved by a third author (ASCO or FBMO).

Data extraction from included studies was done by two reviewers (AFC and LVS) using a Microsoft® Word (Microsoft 365 for Windows) data extraction sheet. Any disagreements in individual judgments of the reviewers were resolved by a third author (ASCO or FBMO). The spreadsheet was composed of five parts:

1. General information about the study i.e. title, authors, year of publication, journal, volume, issue, country and original language.
2. Information about study eligibility i.e. type of study, type of subjects, index test, comparator tests, reference standard, and year of publication.
3. Information about population and setting i.e. population description, number of enrolled subjects, number of subjects who completed the study, excluded subjects, age range, and setting.
4. Information about methods i.e. aim of study, design, method of patient recruitment, duration of participation in the study, statistical analysis, sample collection technique, index test, cut-off value details, comparator test details, reference standard, patient follow-up.
5. Information about study results i.e. index test results, comparison between index test and comparator tests and outcomes.

### Reporting bias assessment

Potentially eligible studies were fully assessed by two reviewers independently (AFC and LVS) with the Quality Assessment of Diagnostic Accuracy Studies (QUADAS-2) tool for judgment of bias and concerns regarding applicability of individual studies (Whiting *et al*., 2011). Any disagreements in individual judgments of the reviewers were resolved by a third author (ASCO or FBMO). Graph preparation was performed in the Review Manager (RevMan, Cochrane Collaboration) version 5.3. software.

### Synthesis method

A narrative synthesis was performed evaluating semen quality parameters in different situations related to psychological stress. Study characteristic results were summarized within the text or in table form. Data was used at an aggregate level and not as individual participants. Descriptive statistics were used to report general characteristics of the included studies. A meta-analysis was not performed in this review due to the heterogeneity of different questionnaires used to assess psychological stress.

## RESULTS

### Study selection

**Figure 1:**
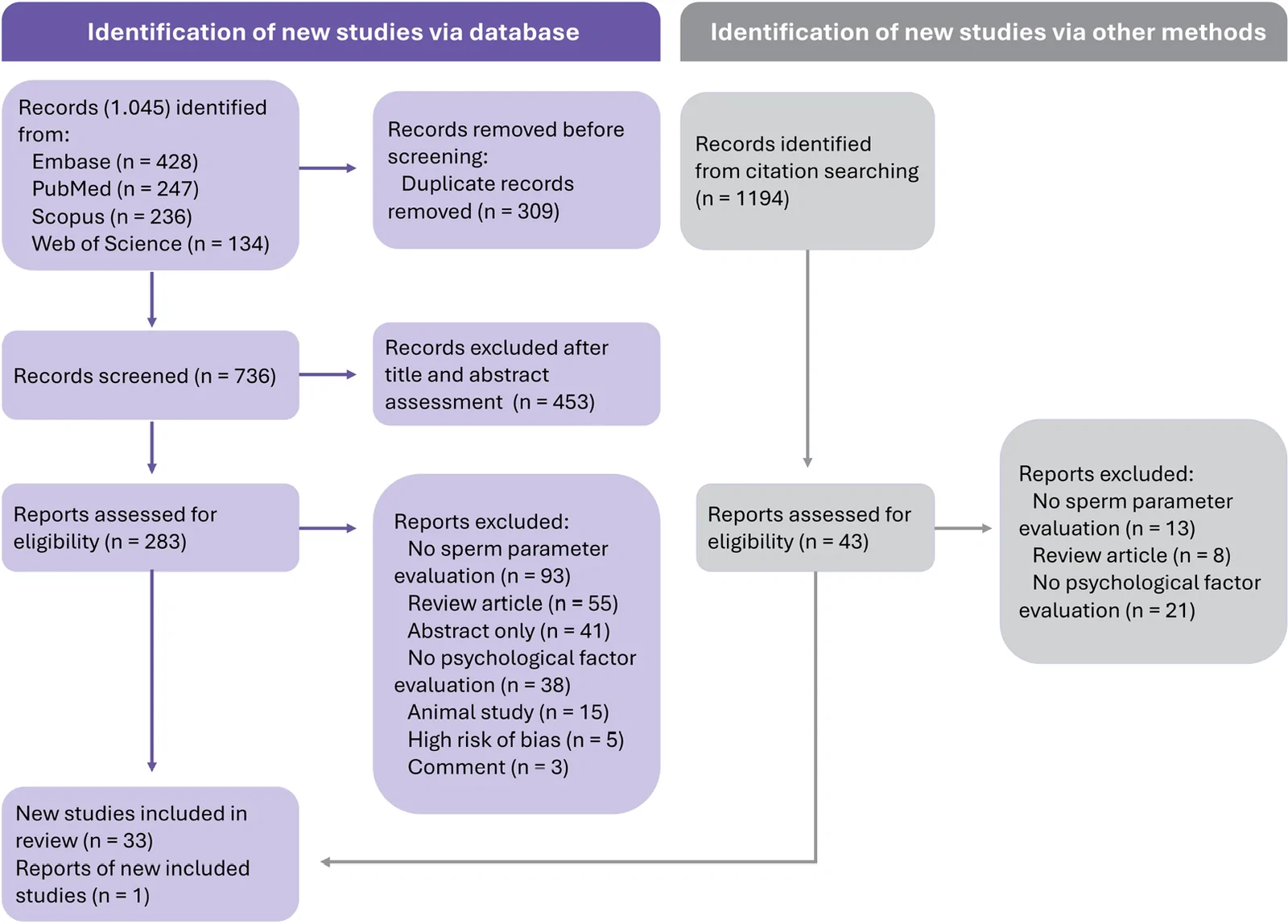
Flow chart of literature search.

Our search strategy retrieved 1,045 records via database, of which 736 were unduplicated. Of these records, 428 were from Embase, 247 were from PubMed, 236 were from Scopus and 134 were from Web of Science. After the first screening, 283 reports were selected as potentially relevant for full-text analysis. After eligibility assessment, 250 reports were excluded from the review because they failed to attend to all items required by the eligibility criteria or presented high-risk of bias. In total, 33 studies were included in our systematic review via database search. Additionally, we identified 1,194 records from the citation of the selected studies, of which 43 were selected for full-text analysis. After eligibility assessment, one study was included in the review, thus totaling 34 studies included in our systematic review. The flow diagram with detailed search process is demonstrated in Figure 1.

### Quality assessment of included studies

**Figure 2:**
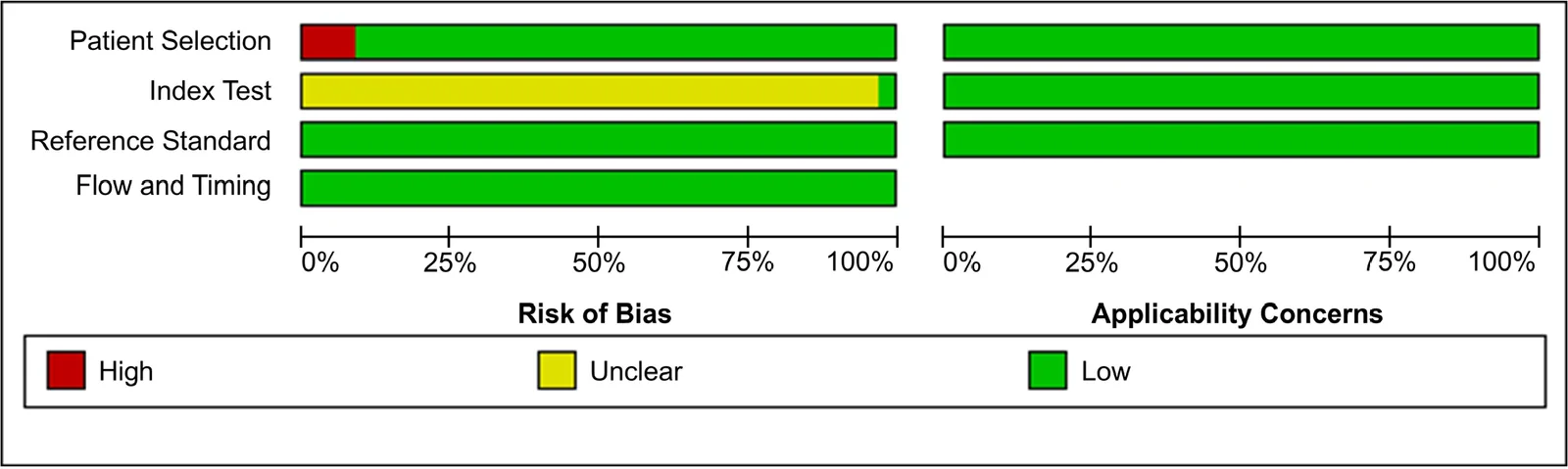
Risk of bias and applicability concerns summary: review authors’ judgements about each domain for each study included

**Figure 3:**
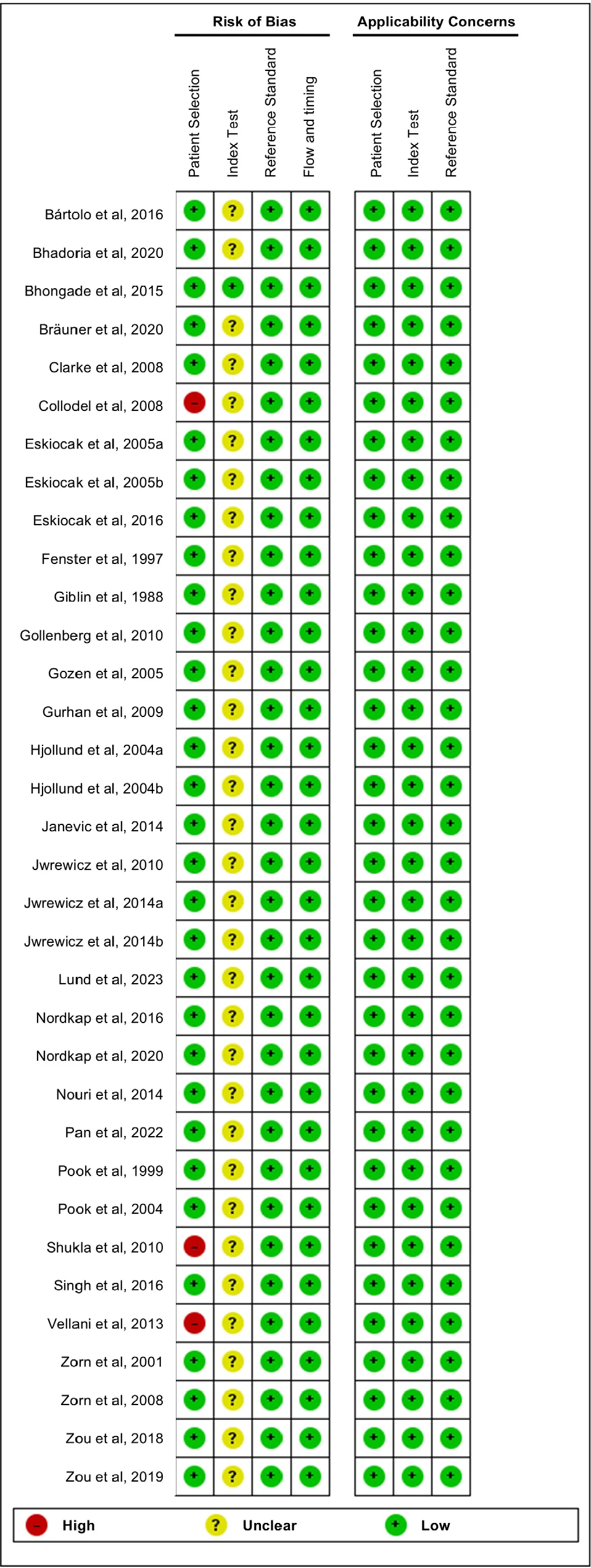
Risk of bias and applicability concerns graph: review authors’ judgements about each domain presented as percentages across included studies.

Risk of bias and applicability concerns are summarized in Figure 2 and Figure 3. Three (8.82%) out of 34 studies included in the review were judged to be at a high risk of bias due to a case-control design assessed in the patient selection section. Except for one study (Bhongade *et al*., 2015) that clearly stated that the person assessing the questionnaire was not aware of the data of semen parameters, all other studies presented unclear risk of bias in the index test section because it was unclear if knowledge of semen parameters could have introduced bias during the processing of participant’s interview answers. Since all studies described thresholds to classify different levels of psychological stress for the questionnaires, the index test category of the QUADAS-2 tool was not considered to be at a high risk of bias because of the uncertainty. All 34 studies met applicability criteria to answer the review question.

### Study characteristics

Included studies were published between 1988 and 2023 and collected a total of 11,141 participants, ranging from 20 to 1,362 participants per study. Eighteen studies had a cross-sectional design (Bártolo *et al*., 2016; Bhongade *et al*., 2015; Bräuner *et al*., 2020; Fenster *et al*., 1997; Gollenberg *et al*., 2010; Janevic *et al*., 2014; Jurewicz *et al*., 2010, 2014a, 2014b; Lund *et al*., 2023; Nordkap *et al*., 2016, 2020; Pan *et al*., 2022; Vellani *et al*., 2013; Wang *et al*., 2022; Zorn *et al*., 2001; Zou *et al*., 2018, 2019); while 16 were longitudinal studies (Bhadoria *et al*., 2020; Clarke *et al*., 1999; Collodel *et al*., 2008; Eskiocak *et al*., 2005a, 2005b, 2006; Giblin *et al*., 1988; Gözen *et al*., 2005; Gürhan *et al*., 2009; Hjollund *et al*., 2004a, 2004b; Nouri *et al*., 2014; Pook *et al*., 1999, 2004; Shukla *et al*., 2010; Zorn *et al*., 2008). Fertile men were investigated in 15 reports (Eskiocak *et al*., 2005a, 2005b, 2006; Fenster *et al*., 1997; Giblin *et al*., 1988; Gollenberg *et al*., 2010; Gözen *et al*., 2005; Gürhan *et al*., 2009; Hjollund *et al*., 2004a, 2004b; Janevic *et al*., 2014; Nordkap *et al*., 2016, 2020; Zou *et al*., 2018, 2019), infertile men in 15 reports (Bártolo *et al*., 2016; Bhadoria *et al*., 2020; Bhongade *et al*., 2015; Clarke *et al*., 1999; Collodel *et al*., 2008; Jurewicz *et al*., 2014a, 2014b; Lund *et al*., 2023; Nouri *et al*., 2014; Pan *et al*., 2022; Pook *et al*., 1999, 2004; Singh *et al*., 2016; Zorn *et al*., 2001, 2008) and from 4 reports we included both fertile and infertile men (Bräuner *et al*., 2020; Jurewicz *et al*., 2010; Shukla *et al*., 2010; Vellani *et al*., 2013). Six studies were performed in Denmark (Bräuner *et al*., 2020; Hjollund *et al*., 2004a, 2004b; Lund *et al*., 2023; Nordkap *et al*., 2016, 2020); five in Turkey (Eskiocak *et al*., 2005a, 2005b, 2006; Gözen *et al*., 2005; Gürhan *et al*., 2009); five in the United States of America (USA) (Clarke *et al*., 1999; Fenster *et al*., 1997; Giblin *et al*., 1988; Gollenberg *et al*., 2010; Janevic *et al*., 2014); four in India (Bhadoria *et al*., 2020; Bhongade *et al*., 2015; Shukla *et al*., 2010; Singh *et al*., 2016); three in Polland (Jurewicz *et al*., 2010, 2014a, 2014b); three in China (Pan *et al*., 2022; Zou *et al*., 2018, 2019); two in Germany (Pook *et al*., 1999, 2004); two in Italy (Collodel *et al*., 2008; Vellani *et al*., 2013); two in Slovenia (Zorn *et al*., 2001, 2008); one in Austria (Nouri *et al*., 2014) and one in Portugal (Bártolo *et al*., 2016). Nearly all studies were published in English, except for one paper published in French (Zorn *et al*., 2001), one in Polish (Jurewicz *et al*., 2010) and another one in Turkish (Gözen *et al*., 2005).

A variety of questionnaires were applied in the studies to assess psychological stress. While some questionnaires were used exclusively by a research group like the Fertility Problem Inventory Questionnaire (Nouri *et al*., 2014), the Self-Administered 7-Item Generalized Anxiety Disorder (GAD-7) Scale (Pan *et al*., 2022) and the General Health Questionnaire (Hjollund *et al*., 2004b), other questionnaires were applied more frequently throughout reports like the State Trait Anxiety Inventory in nine studies (Bártolo *et al*., 2016; Clarke *et al*., 1999; Eskiocak *et al*., 2005a, 2005b, 2006; Gözen *et al*., 2005; Gürhan *et al*., 2009; Shukla *et al*., 2010; Vellani *et al*., 2013), the Stressful Life Events Inventory in six studies (Bräuner *et al*., 2020; Fenster *et al*., 1997; Giblin *et al*., 1988; Gollenberg *et al*., 2010; Janevic *et al*., 2014; Nordkap *et al*., 2020), the Perceived Stress Scale in five studies (Bhadoria *et al*., 2020; Janevic *et al*., 2014; Jurewicz *et al*., 2014a; Lund *et al*., 2023; Nordkap *et al*., 2020), the Job Content Questionnaire in four studies (Fenster *et al*., 1997; Hjollund *et al*., 2004a; Janevic *et al*., 2014; Zou *et al*., 2019) and the Copenhagen Psychosocial Questionnaire in three studies (Bräuner *et al*., 2020; Nordkap *et al*., 2016, 2020). For semen analysis, the 5º edition of the WHO laboratory manual for the examination and processing of human semen was consulted in twelve studies (Bártolo *et al*., 2016; Bhadoria *et al*., 2020; Bhongade *et al*., 2015; Bräuner *et al*., 2020; Jurewicz *et al*., 2014b; Lund *et al*., 2023; Nordkap *et al*., 2016, 2020; Pan *et al*., 2022; Singh *et al*., 2016; Zou *et al*., 2018, 2019), the 4º edition in ten studies (Collodel *et al*., 2008; Gollenberg *et al*., 2010; Janevic *et al*., 2014; Jurewicz *et al*., 2010, 2014a; Pook *et al*., 2004; Shukla *et al*., 2010; Vellani *et al*., 2013; Zorn *et al*., 2001, 2008), the 3^rd^ edition in ten studies (Eskiocak *et al*., 2005b, 2005a, 2006; Fenster *et al*., 1997; Gözen *et al*., 2005; Gürhan *et al*., 2009; Hjollund *et al*., 2004a, 2004b; Nouri *et al*., 2014; Pook *et al*., 1999) and the 1^st^ edition in two studies (Clarke *et al*., 1999; Giblin *et al*., 1988). The 2^nd^ edition of the manual was not used in any of the studies.

Psychological stress exposure was divided into the following categories: academic stress, daily life stress, infertility-related stress, life events stress and work-related stress. Some studies evaluated more than one category in their subjects. Semen quality parameters analyzed in the studies were: sperm volume (SV), sperm concentration (SC), total sperm count (TSP), sperm progressive motility (SPM) and sperm abnormal morphology (SAM).

Semen volume was evaluated in 31 out of 34 studies included in this review. From those 31 studies, four (12.9%) (Giblin *et al*., 1988; Jurewicz *et al*., 2010; Nordkap *et al*., 2016; Vellani *et al*., 2013) showed an association between psychological stress and decreased semen volume. All 34 included assessed sperm concentration and in 18 (52.9%) studies (Bhongade *et al*., 2015; Clarke *et al*., 1999; Eskiocak *et al*., 2005a, 2005b, 2006; Gollenberg *et al*., 2010; Gözen *et al*., 2005; Janevic *et al*., 2014; Nordkap *et al*., 2016, 2020; Pan *et al*., 2022; Pook *et al*., 1999, 2004; Shukla *et al*., 2010; Singh *et al*., 2016; Vellani *et al*., 2013; Zou *et al*., 2018, 2019) an association between psychological stress and decreased sperm concentration was observed. Total sperm count was decreased in stressful situations in nine (56.3%) (Gollenberg *et al*., 2010; Janevic *et al*., 2014; Nordkap *et al*., 2016, 2020; Pan *et al*., 2022; Vellani *et al*., 2013; Zorn *et al*., 2001; Zou *et al*., 2018, 2019) out of 16 studies that evaluated this parameter. Sperm motility was evaluated in 30 out of 34 included studies and an association between decreased sperm motility and psychological stress was observed in 19 (63.3%) (Bártolo *et al*., 2016; Bhongade *et al*., 2015; Clarke *et al*., 1999; Eskiocak *et al*., 2005a, 2005b, 2006; Gollenberg *et al*., 2010; Gözen *et al*., 2005; Gürhan *et al*., 2009; Janevic *et al*., 2014; Jurewicz *et al*., 2010, 2014b; Nordkap *et al*., 2020; Pan *et al*., 2022; Pook *et al*., 1999; Shukla *et al*., 2010; Singh *et al*., 2016; Vellani *et al*., 2013; Zorn *et al*., 2001) studies. Lastly, sperm abnormal morphology was assessed in 28 out of 34 included studies and six (21.4%) (Bhongade *et al*., 2015; Eskiocak *et al*., 2005b; Giblin *et al*., 1988; Gözen *et al*., 2005; Nordkap *et al*., 2016; Singh *et al*., 2016) revealed association with psychological stress. No association between semen parameters and psychological stress was observed in 10 (29.4%) out of 34 studies included in this review. Study characteristics are summarized in Table 1.

**Table 1:** Characteristics and summary of study results

References	Country	Study design	Study subjects	Sample size	Stress exposure	Questionnaire used for stress assessment	Semen quality parameters analyzed	WHO guideline edition	Impaired semen quality parameters with psychological stress
Bártolo *et al.*, 2016	Portugal	Cross-sectional	Infertile men	112	Infertility-related stress	State Trait Anxiety Inventory	SC, SPM, SAM	5º edition	↓ Slow progressive motility
Bhadoria *et al.*, 2020	Índia	Longitudinal	Infertile men	50	Infertility-related stress	Perceived Stress Scale	SV, SC, SPM, SAM	5º edition	None
Bhongade *et al.*, 2015	India	Cross-sectional	Infertile men	70	Infertility-related stress	Hospital Anxiety and Depression Score Questionnaire	SV, SC, TSC, SPM, SAM	5º edition	↓Sperm concentration, ↓ Sperm motility and ↓ Normal sperm morphology
Bräuner *et al.*, 2020	Denmark	Cross-sectional	Fertile and infertile men	423	Infertility-related stress	Copenhagen Psychosocial Questionnaire; Stressful Life Events inventory	SV, SC, TSC, SPM, SAM	5º edition	None
Clarke *et al.*, 1999	USA	Longitudinal	Infertile men	31	Infertility-related stress	State Trait Anxiety Inventory	SV, SC, TSC, SPM, SAM	1º edition	↓Sperm concentration and ↓ Sperm motility
Collodel *et al.*, 2008	Italy	Longitudinal	Infertile men	20	Infertility-related stress	Psychological State of Stress Measure	SV, SC, SPM	4º edition	None
Eskiocak *et al*., 2005	Turkey	Longitudinal	Fertile men	27	Academic stress	State Trait Anxiety Inventory	SV, SC, SPM, SAM	3º edition	↓Sperm concentration, ↓ Sperm motility
Eskiocak *et al*., 2005	Turkey	Longitudinal	Fertile men	34	Academic stress	State Trait Anxiety Inventory	SV, SC, SPM, SAM	3º edition	↓Sperm concentration, ↓ Sperm motility, ↑ Abnormal sperm morphology
Eskiocak *et al*., 2006	Turkey	Longitudinal	Fertile men	29	Academic stress	State Trait Anxiety Inventory	SV, SC, SPM, SAM	3º edition	↓Sperm concentration, ↓ Sperm motility
Fenster *et al*., 1997	USA	Cross-sectional	Fertile men	164	Work-related stress and life events stress	The Job Content Questionnaire; Stressful Life Events Inventory	SV, SC, SPM	3º edition	None
Giblin *et al*., 1988	USA	Longitudinal	Fertile men	28	Daily life stress and life events stress	Van Atta’s Life Stress Questionnaire; Stressful Life Events Inventory	SV, SC, TSC, SPM, SAM	1º edition	↓Volume and ↓ Normal sperm morphology
Gollenberg *et al*., 2010	USA	Cross-sectional	Fertile men	744	Life events stress	Stressful Life Events Inventory; Social Readjustment Rating Scale	SV, SC, TSC, SPM, SAM	4º edition	↓Sperm concentration, ↓ Sperm motility and ↓ Total sperm count
Gözen *et al*., 2005	Turkey	Longitudinal	Fertile men	36	Academic stress	State Trait Anxiety Inventory	SV, SC, SPM, SAM	3º edition	↓Sperm concentration, ↓ Sperm motility and ↓ Normal sperm morphology
Gürhan *et al*., 2009	Turkey	Longitudinal	Fertile men	80	Infertility-related stress	State Trait Anxiety Inventory; Beck Depression Inventory	SV, SC, SPM, SAM	3º edition	↓ sperm motility
Hjollund *et al*., 2004a	Denmark	Longitudinal	Fertile men	399	Work-related stress	The job Content Questionnaire	SV, SC, SAM	3º edition	None
Hjollund *et al*., 2004b	Denmark	Longitudinal	Fertile men	418	Daily life stress	The General Health Questionnaire	SV, SC, SAM	3º edition	None
Janevic *et al*., 2014	USA	Cross-sectional	Fertile men	193	Work-related stress, life events and daily life stress	The Job Content Questionnaire; Perceived Stress Scale; Stressful Life Events Inventory	SV, SC, SPM, SAM	4º edition	↓Sperm concentration, ↓ Sperm motility and ↓ Total sperm count
Jurewicz *et al*., 2010	Polland	Cross-sectional	Fertile and infertile men	179	Work-related stress	Subjective Work Characteristics Questionnaire	SV, SC, SPM, SAM	4º edition	↓ Semen volume ↓ Sperm motility
Jurewicz *et al*., 2014a	Polland	Cross-sectional	Infertile men	336	Work-related stress	Exposure to Occupational Factors Questionnaire	SV, SC, SPM, SAM	5º edition	↓ Sperm motility
Jurewicz *et al*., 2014b	Polland	Cross-sectional	Infertile men	327	Work-related stress and daily life stress	Subjective Work Characteristics Questionnaire; Perceived Stress Scale	SV, SC, SPM, SAM	4º edition	None
Lund *et al*., 2023	Denmark	Cross-sectional	Infertile men	1,159	Infertility-related stress	Perceived stress scale	SV, SC, TSC	5º edition	None
Nordkap *et al*., 2016	Denmark	Cross-sectional	Fertile men	1,215	Daily life stress	The Copenhagen Psychosocial Questionnaire	SV, SC, TSC, SPM, SAM	5º edition	↓Semen volume, ↓Sperm concentration, ↓ Total sperm count, ↓ Normal sperm morphology
Nordkap *et al*., 2020	Denmark	Cross-sectional	Fertile men	1,362	Daily life stress and life events stress	The Copenhagen Psychosocial Questionnaire; Stressful Life Events Inventory; Perceived Stress Scale	SV, SC, TSC, SPM, SAM	5º edition	↓Sperm concentration, ↓ Total sperm count, ↓ Sperm motility
Nouri *et al*., 2014	Austria	Longitudinal	Infertile men	78	Infertility-related stress	Fertility Problem Inventory Questionnaire	SV, SC, TSC, SPM	3º edition	None
Pan *et al*., 2022	China	Cross-sectional	Infertile men	378	Infertility-related stress	Self-Administered 7-Item Generalized Anxiety Disorder (GAD-7) Scale	SV, SC, TSC, SPM, SAM	5º edition	↓Sperm concentration, ↓ Total sperm count, ↓ Sperm motility
Pook *et al*., 1999	Germany	Longitudinal	Infertile men	158	Infertility-related stress	Infertility Distress Scale	SC, SPM, SAM	3º edition	↓Sperm concentration and ↓ Sperm motility
Pook *et al*., 2004	Germany	Longitudinal	Infertile men	120	Infertility-related stress	Infertility Distress Scale	SC	4º edition	↓Sperm concentration
Shukla *et al*., 2010	India	Longitudinal	Fertile and infertile men	120	Infertility-related stress	State Trait Anxiety Inventory	SV, SC, TSC, SPM	4º edition	↓Sperm concentration and ↓ Sperm motility
Singh *et al*., 2016	India	Cross-sectional	Infertile men	80	Infertility-related stress	Hospital Anxiety and Depression Score questionnaire	SV, SC, TSC, SPM, SAM	5º edition	↓Sperm concentration, ↓ Sperm motility and ↓ Normal sperm morphology
Vellani *et al*., 2013	Italy	Cross-sectional	Fertile and infertile men	179	Infertility-related stress	State Trait Anxiety Inventory	SV, SC, TSC, SPM, SAM	4º edition	↓Sperm volume, ↓Sperm concentration, ↓ Total sperm count, and ↓ Sperm motility
Zorn *et al*., 2001	Slovenia	Cross-sectional	Infertile men	495	Infertility-related stress	Zung's Anxiety Scale Inventory questionnaire	SV, SC, TSC, SPM, SAM	4º edition	↓ Total sperm count, and ↓ Sperm motility
Zorn *et al*., 2008	Slovenia	Longitudinal	Infertile men	1076	Infertility-related stress	Zung's Anxiety Scale Inventory questionnaire	SV, SC, SPM, SAM	4º edition	None
Zou *et al*., 2018	China	Cross-sectional	Fertile men	637	Daily life stress	Self-rating Depression Scale	SV, SC, TSC, SPM, SAM	5º edition	↓Sperm concentration, ↓ Total sperm count,
Zou *et al*., 2019	China	Cross-sectional	Fertile men	384	Work-related stress	The job Content Questionnaire	SV, SC, TSC, SPM, SAM	5º edition	↓Sperm concentration, ↓ Total sperm count,

Abbreviations: total sperm count (TSP), sperm concentration (SC), sperm progressive motility (SPM), sperm abnormal morphology (SAM) and sperm vitality (SV); World Health Organization (WHO).

### Stress categories

Stress categories were divided into 5 subgroups. Academic stress was evaluated in 4 studies, daily life stress in 7, infertility-related stress in 17, life events stress in 5 and work-related stress in 7 studies. Association between stress categories and impaired semen quality parameters is presented on table 2.

**Table 2:** Association between stress categories and impaired semen quality parameters

Stress category	Total number of studies	Impaired semen quality parameters number of studies (%)					No association number of studies (%)
		Volume	Total sperm count	Sperm concentration	Sperm progressive motility	Sperm abnormal morphology	
Infertility-related stress	17	1 (5.9%)	3 (17.7%)	8 (47.1%)	10 (58.8%)	2 (11.8%)	6 (42.9%)
Academic stress	4	-	-	4 (100%)	4 (100%)	2 (50.0%)	-
Work-related stress	7	1 (14.3%)	2 (28.6%)	2 (28.6%)	3 (42.9%)	-	3 (42.9%)
Life events stress	5	1 (20.0%)	3 (60.0%)	3 (60.0%)	3 (60.0%)	1 (20.0%)	1 (20.0%)
Daily life stress	7	2 (28.6%)	4 (57.1%)	4 (57.1%)	2 (28.6%)	2 (28.6%)	3 (42.9%)

Semen volume was evaluated in 4 categories, except for the academic stress category. It showed poor association with perceived stress overall with a maximum of 28.6% for the daily life stress category. Sperm abnormal morphology was also evaluated in 4 categories, except for the work-related stress category. It showed less consisted association with perceived stress, ranging from 28.6% in the daily life stress category to 50.0% in the academic stress category. Total sperm count was also inconsistently affected by perceived stress, ranging from 17.7% in the infertility-related stress category to 60.0% in the life events category. Sperm concentration and sperm progressive motility were evaluated in all stress categories and were more consistently associated with perceived stress. Sperm concentration was affected to a minimum of 28.6% by work-related stress, but to a maximum of 100% by academic stress. Likewise, sperm progressive motility was affected to a minimum of 28.6% by daily life stress to a maximum of 100% by academic stress.

## DISCUSSION

### Academic stress

During college, students face unrivaled levels of distress when experiencing new life situations that can affect their mental health (Liu *et al*., 2019). In this review, four studies evaluated psychological stress in the academic environment (Eskiocak *et al*., 2005a, 2005b, 2006; Gözen *et al*., 2005). These studies were performed in Turkey and shared a few similarities like application of the same questionnaire to assess psychological stress (State Trait Anxiety Inventory) and the use of the 3^rd^ edition of the WHO manual to assess semen parameters. Additionally, participants in all four studies were medical students who were assessed for psychological stress before (stress period) and after (non-stress period) their final university examinations. Eskiocak *et al* (2005a) found that spermatozoa concentrations, motility index and percentage of rapid progressive motility decreased under stress. The same research group also published in 2005 that in the stress condition of final exams, density of spermatozoa, percentage of total progressive and rapid progressive motile spermatozoa were significantly lower than those found under non-stress conditions, whereas the percentage of immotile spermatozoa and abnormal morphology were increased (Eskiocak *et al*., 2005b). Gözen *et al.* (2005) published that sperm count, percentage of progressive motility and percentage of normal morphology significantly decreased during stress period. Eskiocak *et al*. (2006) observed that during stressful periods, sperm concentration and rapid progressive motility were also significantly lower than in non-stressful situations. These studies revealed that a stressor experienced in college may have an impact on sperm health.

### Daily life stress

Daily stress is defined as mundane hassles, strains, or annoyances associated with daily activities that have potential to disturb the routine and add stress to a person’s life (Sweeney & Upchurch, 2013). Daily life stress was evaluated in seven studies (Giblin *et al*., 1988; Hjollund *et al*., 2004b; Janevic *et al*., 2014; Jurewicz *et al*., 2014a; Nordkap *et al*., 2016, 2020; Zou *et al*., 2018). Janevic *et al.* (2014) and Nordkap *et al*. (2020) observed an inverse association between perceived stress score and sperm concentration, motility, and morphology with the usage of the Cohen Perceived Stress Scale in fertile men. Jurewicz *et al*. (2014a) also applied the Cohen Perceived Stress Scale in their subjects, but there was no correlation between the level of perceived life stress and semen quality indicators in the study. Similarly, the study by Hjollund *et al*. (2004b), found that semen quality was not related to the psychological questionnaire score using the General Health Questionnaire.

Besides applying the Cohen Perceived Stress Scale, Nordkap *et al*. (2020) also interviewed subjects with the Copenhagen Psychosocial Questionnaire and inferred those men with the highest stress symptom scores had lower sperm concentration, total sperm count and motility. In 2016, the same research group also observed a negative association between self-reported stress using the Copenhagen Psychosocial Questionnaire and semen quality, in which men with stress scores above the reference level had significantly lower semen volume, sperm concentration, total sperm count, and total number of morphologically normal spermatozoa (Nordkap *et al*., 2016). Applying the Self-rating Depression Scale, Zou *et al*. (2018) observed that men with high depression scores had lower sperm concentration and total sperm count than non-depressed men. Giblin *et al*. (1988) verified that volume and percentage of normal morphologic features were negatively associated with self-reported stress symptoms using the Van Atta’s Life Stress Questionnaire.

### Infertility-related stress

Psychological stress has been clinically perceived as a potential risk factor for male infertility, whereas stress related to infertility is associated with a poorer fertility treatment outcome in men (Boivin & Schmidt, 2005; Ilacqua *et al*., 2018). The most used tool to assess psychological stress in men with infertility-related stress was the State Trait Anxiety Inventory, which was applied in five studies (Bártolo *et al*., 2016; Clarke *et al*., 1999; Gürhan *et al*., 2009; Shukla *et al*., 2010; Vellani *et al*., 2013). In a study with infertile men, Shukla *et al*. (2010) observed that stress scores, elaborated based on the questionnaire, were found significantly high in infertile subjects. Sperm concentration and motility in all stressed infertile groups as compared with controls were found decreased. In patients undergoing In Vitro Fertilization (IVF) procedures, Vellani *et al.* (2013) found that increased levels of both state and trait anxiety were associated with lower semen volume, sperm concentration and count, and reduced sperm motility. In a similar way, Clarke *et al.* (1999) study objective was to determine the relationship between psychological stress and semen quality among men undergoing IFV for the first time at a pre-IVF sampling period and at the time of egg retrieval. They observed that scores were significantly and inversely correlated with changes in several sperm at the time of oocyte retrieval, including total sperm concentration, motile sperm concentration, and total motile spermatozoa. On the other hand, Gürhan *et al*. (2009) found no significant relation for sperm counts with anxiety scores on the day of egg retrieval, although sperm motility was weakly and inversely correlated with depression scores assessed with the Beck Depression Inventory. Still applying the State Trait Anxiety Inventory to assess psychological stress, Bártolo *et al*. (2016) observed that anxiety had a negative linear impact on the slow progressive motility of men undergoing first experience Assisted Reproductive Technology (ART) treatments, but not on men on repeated ART cycles.

Parameters like sperm count, motility and morphologically normal spermatozoa were lower in male partners of infertile couples who scored higher in the Hospital Anxiety and Depression Score Questionnaire in the studies performed by Bhongade *et al*. (2015) and Singh *et al*. (2016). Pan *et al.* (2022) performed a multicenter study in North China to evaluate the association between generalized anxiety symptoms and semen quality in infertile men applying the Self-Administered 7-Item Generalized Anxiety Disorder (GAD-7) Scale. They verified that individuals with abnormal GAD-7 scale scores had a significantly lower sperm count, sperm concentration, and progressive motility than those with normal GAD-7 scale scores. The Infertility Distress Scale was applied by Pook *et al*. in studies published in 1999 (Pook *et al*., 1999) and 2004 (Pook *et al*., 2004). The first study was performed to verify whether stress had a negative influence on semen quality parameters. It was observed that infertility distress was higher in men with declining sperm concentration and motility compared to those with improving parameters (Pook *et al*., 1999). The second study sought to deliver evidence for a negative impact of distress due to infertility on sperm concentration. They concluded that while the mean sperm concentration was stable in the group of non-seriously distressed patients, there was a decline of 33.3% in the group of highly distressed patients (Pook *et al*., 2004).

Zorn et al used the Zung’s Anxiety Scale Inventory questionnaire in studies published in 2001 (Zorn *et al*., 2001) and 2008 (Zorn *et al*., 2008) to evaluate male partners of infertile couples. In 2001, they observed that an intense reaction to chronic stress was associated with a decreased sperm count and decrease progressive motility, while exposure to acute stress was associated with increased progressive motility (Zorn *et al*., 2001). In the study published in 2008 no correlation was found between psychological factors and sperm rapid progressive motility and normal morphology, although poor coping with stress was associated with increased occurrence of early miscarriage. (Zorn *et al*., 2008).

Both Bhadoria *et al*. (2020) and Lund *et al*. (2023) used the Cohen Perceived Stress Scale to assess psychological stress in men with infertility-related stress and neither found an association between semen parameters and stress. Likewise, Bräuner *et al*. (2020) found no differences in psychological stress symptoms between fertile and infertile men applying the Copenhagen Psychosocial Questionnaire. Use of the Fertility Problem Inventory Questionnaire by Nouri *et al.* (2014) also did not find an association between stress and the decline in semen quality during in vitro fertilization. In a study by Collodel *et al.* (2008), patients with idiopathic infertility were selected after evaluation of psychological stress to evaluate a positive effect of a stress therapy on their semen quality. Although the number of “healthy” sperm was significantly higher in the treated group after therapy, indicating a recovery of sperm quality, no significant decrease in sperm pathologies was observed.

### Life events stress

There is a close link between stressful life events and physical health, although susceptibility to stress varies from person to person (Salleh, 2008; Tosevski & Milovancevic, 2006). Psychological stress due to stressful life events were assessed in five studies using mainly the Holmes and Rahe’s Social Readjustment Scale (Bräuner *et al*., 2020; Fenster *et al*., 1997; Gollenberg *et al*., 2010) or the Cochrane Life Events Inventory (Giblin *et al*., 1988; Janevic *et al*., 2014). Three studies (Bräuner *et al*., 2020; Fenster *et al*., 1997; Giblin *et al*., 1988) observed that total number of stressful life events were not associated with reduced male reproductive function, although Fenster *et al*. (1997) observed that recent death of a close family member was associated with a reduction in percent of progressively motile sperm. Gollenberg *et al.* (2010) found that experience of two or more life events was associated with decreased sperm concentration, total count, and percent motile sperm. Furthermore, Janevic *et al.* (2014) observed that participants who reported two or more stressful life events in the past year had a lower percentage of motile sperm and a lower percentage of morphologically normal sperm, but a similar sperm concentration.

### Work-related stress

Work-related stress was evaluated with the Job Content Questionnaire based on Karasek’s model in four studies (Fenster *et al*., 1997; Hjollund *et al*., 2004a; Janevic *et al*., 2014; Zou *et al*., 2019). In a study published by Zou *et al.* (2019), men with high work stress had a higher risk of sperm concentration and total sperm count being classified below the WHO thresholds than men with lower work stress. On the other hand, stress at work was not related to differences in semen quality in the studies published by Fenster *et al*. (1997), Hjollund *et al*. (2004a) and Janevic *et al*. (2014).

Work-related stress was also assessed by a Polish research group with one publication in 2010 (Jurewicz *et al*., 2010) and two in 2014 (Jurewicz *et al*., 2014a, 2014b). Jurewicz *et al*. (2010) considered the sum of points from the Subjective Work Assessment Questionnaire received by respondents and found a significant negative impact only on the percentage of progressive motility. Using the same questionnaire, Jurewicz *et al*. (2014a) found a positive association between stress and the percentage of atypical sperm, but no association with other semen quality indicators. By using the Exposure to Occupational Factors Questionnaire, another publication by Jurewicz *et al*. (2014b) found that exposure to noise during work was associated with decreased motility.

### Biological mechanism

The primary biological link between psychological stress and diminished semen quality involves the disruption of the hypothalamic-pituitary-gonadal (HPG) axis. HPG axis finely tunes male reproductive function through a series of hormonal and neuroendocrine signals. The hypothalamus, which is linked to central nervous system regions like the limbic system, coordinates responses to stress. When a person experiences acute or chronic stress, the resulting fluctuations in stress-related hormones disrupt the normal signaling of the HPG axis. This hormonal alteration subsequently modifies the function of Sertoli cells and their associated blood-testis barrier, ultimately impairing spermatogenesis and semen quality (Bhongade *et al.*, 2015; Nargund, 2015; Qiu *et al.*, 2023). Additionally, cortisol, a steroid hormone released during acute and chronic stress and implicated in various physical and mental disorders, has been shown to interfere with male fertility by disrupting the function of the CatSper channel. This sperm-specific, calcium-selective cation channel, located in the principal piece of the sperm tail, is essential for fertility as it regulates the calcium influx necessary for processes like hyperactivation of motility, chemotaxis, capacitation, and the acrosome reaction (Singh and Rajender, 2015). It has been demonstrated that hydrocortisone, the synthetic form of cortisol, disrupts progesterone signaling at CatSper, competitively interfering with the progesterone-induced calcium influx required for proper sperm activation. This mechanistic interaction provides a biological explanation for the observed negative association between anxiety symptoms and reduced progesterone-induced acrosome reaction, suggesting that stress-related increases in cortisol may impair fertility by antagonizing progesterone’s action on CatSper (Sánchez González *et al.*, 2023). Along with calcium influx, reactive oxygen species (ROS) also play an important role in sperm maturation and fertilization capacity. High concentration of polyunsaturated fatty acids in the sperm plasma membrane is essential for its fluidity but also makes the sperm highly susceptible to oxidative damage. Oxidative stress occurs when the levels of ROS in the semen overwhelm the protective capacity of antioxidants. This stress negatively impacts fertility by inducing premature capacitation and compromising the integrity of the sperm plasma membrane, thereby making the sperm less capable of successful fertilization (Aitken, 2017; Agarwal *et al.*, 2018).

### Clinical implications

The link between the ability to procreate and virility may influence men’s reactions to infertility, to the extent that men may perceive the inability to father a child as a threat to their self-image of masculinity (Culley *et al.*, 2013). Furthermore, men are less likely than women to seek psychological help due to the perceived traditional male gender role, which includes independence, self-control, and self-sufficiency (Pederson & Vogel, 2007). It has been verified that men undergoing infertility treatment experience similar levels of distress as women (Peronace et al., 2007). Men participating in online support groups were able to openly discuss a range of feelings and concerns regarding the infertility experience. Instead of simply being “disappointed” about their inability to become fathers, these men were clearly experiencing a range of negative emotions and difficulties. Online support groups can provide an useful venue and context for men to open up about their fertility issues, without the inhibitions associated with face-to-face discussions (Richard *et al.*, 2017).

### LIMITATIONS

The main limitation observed in this review was the lack of homogeneity to measure psychosocial stress between studies. In total, 18 different questionnaires were applied throughout studies, making it arduous to compare results. We also could not directly compare the same tools used to assess psychological stress because of different study designs and study populations Thus, the ability to draw robust conclusions was severely restricted by significant heterogeneity across the reviewed literature. The predominance of cross-sectional designs and the risk of reporting bias in self-assed stress measures was also a limiting factor. Furthermore, the use of different editions of the WHO laboratory manual for the examination and processing of human semen have also contributed to making comparability between studies even more difficult. Cultural variation in stress perception and reporting could also introduce bias to the analysis since studies from 11 countries were included. It has been observed that stress is more common in high-income countries than in lowand middle-income countries (Smith and Wesselbaum, 2025).

## CONCLUSIONS

Sources of psychological stress such as academic stress, daily life stress, infertility-related stress, stressful life events, and work-related stress were correlated to impairment of semen parameters in 70.6% of included studies. Psychological stress is frequently associated with impaired semen parameters, particularly sperm motility, total sperm count, and sperm concentration. Nevertheless, these findings should be interpreted with caution. Most of the reported associations are observational and do not establish a definitive causal relationship between psychological stress and semen quality impairment. Further well-designed, standardized research is needed to clarify the nature and direction of these associations.
